# The 2014 Beatson International Cancer Conference: Powering the Cancer Machine

**DOI:** 10.1186/2049-3002-2-25

**Published:** 2014-11-28

**Authors:** Jurre J Kamphorst, Daniel J Murphy

**Affiliations:** Cancer Research UK Beatson Institute & Institute of Cancer Sciences, University of Glasgow, Garscube Estate, Switchback Road, Glasgow, G61 1BD UK

**Keywords:** Cancer metabolism, Metabolic stress, Metabolic signalling

## Abstract

Here, we present a report of the 2014 annual **Beatson International Cancer Conference**, Glasgow, July 6–9, 2014. The theme was “Powering the Cancer Machine”, focusing on oncogenic signals that regulate metabolic rewiring and the adaptability of the metabolic network in response to stress.

## Opening session

Isocitrate dehydrogenase (IDH) mutations are found in a variety of cancers including acute myeloid leukaemia. IDH1 normally interconverts isocitrate and α-ketoglutarate (αKG). The mutant isoform, expressed in a subset of gliomas and acute myeloid leukaemias, instead converts isocitrate to the non-degradable 2-hydroxyglutarate (2HG). William Kaelin (Dana-Farber Cancer Institute) showed that expression of the R132H mutant IDH1 drives growth factor-independent proliferation of TF-1 leukaemia cells, which are otherwise dependent on GM-CSF. He further demonstrated that (R)-2HG, the enantiomer produced by the R132H IDH mutant, could also support GM-CSF-independent growth. It was found that (R)-2HG and (S)-2HG differentially interact with a class of enzymes that use αKG as a co-factor, including JmjC, Tet, PHD and EglN family proteins, through which they elicit epigenetic alterations and affect hypoxia-inducible factor (HIF) levels.

The mTOR pathway represents a central cellular nutrient-sensing hub, which controls many aspects of cellular metabolism. The tuberous sclerosis (TSC) tumour suppressor proteins (TSC1 and TSC2) are inhibitors of mTOR activation. Michael Hall (University of Basel) showed that deletion of TSC1 in the liver drives mTORC1 hyperactivation, resulting in elevated secretion of FGF21, a reduction in body temperature and reduced nocturnal activity. Treatment of mice with rapamycin lowered FGF21 levels and restored normal body temperature and activity levels. These studies represent a novel observation that mTOR activation could reduce body temperature, while previous studies have shown that mTOR inhibition by rapamycin could affect body size and prolong lifespan.

David Sabatini (Whitehead Institute, MIT), who discovered mTOR while a graduate student with Solomon Snyder at Johns Hopkins, discussed cell-autonomous regulation of TORC1 and focused specifically on amino acid sensing by the TORC1 pathway. In the presence of amino acids, TORC1 is recruited to lysosomes via Rag GTPase heterodimers. However, neither the Rag proteins themselves nor their guanine nucleotide exchange factor, Ragulator, directly bind to amino acids. The amino acid-sensing component of the pathway likely resides within the lysosome, where amino acids are concentrated up to millimolar levels.

## Metabolic signalling

It has been documented that MYC drives the Warburg effect, which describes the propensity for cancer cells to import large amounts of glucose and excrete most of it as lactate. Lactate is transported through monocarboxylate transporters (MCTs). John Cleveland (Moffitt Cancer Center) documented that MYC could directly induce the expression of MCT1 (SLC16A1) and discussed the therapeutic potential of MCT1 inhibition in the context of MYC-driven breast cancer and Burkitt’s lymphoma. MCT1 inhibition suppresses activity of the lower half of the glycolytic pathway and glutathione production, resulting in the accumulation of reactive oxygen species (ROS). MCT1 inhibition moreover suppresses the Cori cycle, through which lactate is removed from the circulation by the liver and used to drive gluconeogenesis. Treatment with the MCT1 inhibitor thus lowers circulating glucose and lactate, suggesting an additional use for such agents to treat type II diabetes.

The cell’s energetic state is continuously monitored by the AMP-activated protein kinase AMPK, which responds dynamically to changes in the ATP:AMP ratio in order to suppress anabolism and promote catabolic activity, thereby maintaining energetic homeostasis. Daniel Murphy (University of Glasgow) spoke of exploiting this feedback mechanism as a strategy for targeting MYC in cancer. MYC accelerates ATP consumption, resulting in progressive activation of AMPK. Surprisingly, a related kinase ARK5 (aka NUAK1) is required for efficient activation of AMPK by MYC, and depletion of either is synthetically lethal with MYC overexpression. Inhibition of ARK5 acutely perturbs mitochondrial respiration, and genetic experiments suggest that targeting ARK5 may be effective against some forms of colorectal cancer. Reuben Shaw (Salk Institute) discussed an affinity trap strategy for isolation of physiological targets of AMPK using a SILAC approach adapted for use in vivo and noted that AMPKα1/α2 double-knockout cells also exhibit mitochondrial defects. Consistent with this observation, D. Grahame Hardie (University of Dundee) stressed that promotion of glycolysis by AMPK is an acute phenomenon whereas sustained activation promotes oxidative phosphorylation. As a negative regulator of TORC1-driven cell growth, AMPK is implicated in tumour suppression and may thus serve different roles at different stages of tumour development. Recent reports that AMPK can be activated by binding to ADP, as well as to AMP, were discussed as were arguments against a major contribution by ADP levels to AMPK regulation under physiological conditions.

Owen Sansom (Beatson Institute) discussed perturbations to mTORC1 signal transduction in APC-deleted intestinal epithelium. Loss of APC leads to deregulated β-catenin activity and consequently to elevation of MYC expression. In intestinal epithelium, this correlates with increased mTORC1 activity, as evidenced by elevated levels of phospho-S6K and phospho-4E-BP1, and MYC deletion coincident with APC loss restores mTORC1 activity to normal. Treatment of floxed APC mice with rapamycin reversibly prevents tumour progression in the intestine whereas mutation of KRas in combination with APC loss is associated with resistance to rapamycin.

Increased protein translation in tumour cells generates its own limitations as cells struggle to maintain the required supply of amino acids. Brendan Manning (Harvard University) presented evidence that TORC1 deregulation simultaneously drives increased protein turnover as well as increased protein production and both effects are sensitive to rapamycin. Accordingly, cells with deregulated mTORC1 are selectively sensitive to bortezomib-mediated inhibition of the 40S proteasome. SREBP1 activation downstream of TORC1 drives NRF1-mediated coordinated transcription of proteasome subunits in a pre-programmed adaptive response to anabolic stress.

Macropinocytosis, a process associated with Ras mutation through which cells absorb nutrients in bulk, may reflect another mechanism to replenish amino acid levels. Dafna Bar-Sagi (New York Langone Medical Center) described recent work on the role of macropinocytosis in tumour metabolism. Using a combination of microscopy and isotope tracing, it was found that macropinocytosis enables the consumption and degradation of extracellular protein to support metabolism. Various aspects of the significance of this novel mode of eating were discussed.

In addition to its roles in driving increased protein translation and elongation, John Blenis (Weill Cornell Medical College) presented evidence that TORC1 also regulates splicing efficiency. Downstream of TORC1, recruitment of S6K1, but not S6K2, occurs to the exon junction complex via specific interaction with SKAR (polymerase delta-interacting protein 3). Recruitment of both factors is required for the efficient expression of intron-containing pre-mRNAs.

Reactive Oxygen species (ROS) play a complex role in tumourigenesis being at once mutagenic and cytotoxic, depending on levels. Boudewijn Burgering (UMC, Utrecht) discussed direct sensing of ROS by Forkhead transcription factors via oxidation of cysteine residues, allowing the formation of stable intermolecular disulfide bridges between FOXO and other proteins, such as p300. Treatment of cells with hydrogen peroxide drives FOXO into the nucleus, activating a transcription programme to counteract ROS.

## Metabolic pathways and stress

As reported earlier, HIFs play an important role in tumour progression. While in gliomas and leukaemias HIF levels are affected by 2HG, in clear cell renal cell carcinoma (ccRCC), HIF1α is stabilised by loss-of-function of the Von Hippel-Lindau (VHL) protein. A newly discovered mechanism of HIF activation in ccRCC was presented by Celeste Simon (University of Pennsylvania), who discussed the role of the gluconeogenic enzyme fructose-1,6-bisphosphatase 1 (FBP1). This enzyme is almost uniformly depleted in 600 ccRCC tumours examined. FBP1 opposes aerobic glycolysis in renal tubular epithelial cells. Unexpectedly, FBP1 was also found to inhibit nuclear HIF function in a non-canonical fashion by interacting with its inhibitory domain. Loss of FBP1 therefore may collaborate with the prominent VHL mutations in ccRCC to promote HIF activation and thus tumour growth.

The metabolism of cancer cells is likely affected by the stressful conditions of the tumour microenvironment. Ralph DeBerardinis (University of Texas, Southwestern) therefore emphasised the need for protocols for in vivo isotope tracing. A case study was shown where human glioblastoma (GBM) patients were infused with ^13^C-glucose prior to excision of their tumour and analysis. Using this approach, it was found that in vivo, GBM tumours consistently had surprisingly high pyruvate dehydrogenase and hence citric acid cycle activity, driving net production of glutamate.

The enzyme phosphoglycerate dehydrogenase (PHGDH) catalyses the first step in the conversion of the glycolytic intermediate 3-phosphoglycerate to serine and glycine. PHGDH has been found to be amplified or overexpressed in a variety of cancers, indicating the importance of these amino acids in cancer cell proliferation. While serine and glycine can be interconverted by serine hydromethyl transferases (SHMT 1 and 2), the relative importance of these amino acids in tumour growth remains unclear. Oliver Maddocks (Beatson Institute) showed that only serine rescued reduced proliferation of cancer cells in medium lacking both serine and glycine. This corroborated the finding that most cell lines avidly consume exogenous serine and only uptake glycine when serine depletes. Serine plays an important role in folate metabolism, supplying one-carbon units for purine nucleotide synthesis.

Anne Brunet (Stanford University) discussed the metabolic regulation of aging using the *C. elegans* model. She focused on histone methylation as deficiencies in trimethylation of histone 3 at lysine 4 (H3K4me3) were found to increase lifespan. Regulation of H3K27me3 was also involved in lifespan determination. Brunet further discussed the interesting observation that H3K4me3-deficient worms have high levels of fat and are long-lived, in apparent contrast with the also long-lived but lean worms subjected to dietary restriction. The H3K4me3-deficient worms were found to have particularly high levels of the fatty acids palmitoleic and cis-vaccenic acid. This was found to be due to the high activity of the desaturase FAT-7/SCD1, and hence, it was postulated that this enzyme plays a role in longevity.

Autophagy is a cellular degradation pathway that can, depending on the context, have a tumour-suppressing or tumour-promoting role. Alec Kimmelman (Dana-Farber Cancer Institute) explained how this role of autophagy in cancer depends on both the type and timing of genetic alterations that occur. Recent experiments in a genetically engineered mouse model of pancreatic cancer (PDAC) with oncogenic KRas and homozygous loss of *Trp53* showed that loss of autophagy accelerated tumour progression. Kimmelman showed that in a similar mouse model with instead sporadic LOH of *Trp53*, tumour growth was dependent on autophagy. In this setting, autophagy may promote tumour progression by maintaining metabolic homeostasis during nutrient starvation. Kimmelman further described how specific cargo is targeted for selective autophagy. Nuclear receptor coactivator 4 (NCOA4) was found to be highly enriched in lysosomes where it acts as a selective cargo receptor for turnover of ferritin to sustain iron homeostasis.

Daniel Peeper (Netherlands Cancer Institute) discussed a surprising role for pyruvate dehydrogenase kinase 1 (PDK1) in suppressing oncogene-induced senescence (OIS) in BRaf^V600E^-driven melanoma. Pyruvate dehydrogenase is rate limiting for entry of pyruvate into the TCA cycle and contributes to maintenance of senescence in pre-neoplastic nevi. Forced expression of PDK1 inhibits PDH, bypasses OIS and thereby facilitates progression to melanoma. Importantly, suppression of PDK combines with paclitaxel to drive regression of established melanomas.

## Therapeutic opportunities

Susan Critchlow (AstraZeneca) described efforts to target MCTs. As reported above, MYC induces MCT1 while HIF1 could activate the expression of MCT4. Given that the tumour microenvironment permits the commensal existence of hypoxic cells that export lactate and respiring cells that could import lactate for oxidation, inhibition of MCTs would be of therapeutic interest. Highly glycolytic tumour cells depend on these transporters to export rapidly produced lactate. Inhibition of MCT1 with a novel small molecule (AZD3965) decreased the proliferation rate of Raji Burkitt lymphoma cells, both in vitro and in vivo. These observations are consistent with the work of John Cleveland.

Chi Van Dang (University of Pennsylvania) provided a background on the MYC oncogene and briefly discussed two recent publications from the Amati and Eilers groups in support of the case that MYC does indeed have specific transcriptional targets. He provided a conceptual framework for oncogene-dependent nutrient addiction, reasoning that constitutive activation of growth factor-independent cell growth and proliferation would render cancer cells addicted to nutrients to support deregulated growth. He also showed that MYC-dependent transformation systems are dependent on both glucose and glutamine, hence causing MYC-dependent cancers to be sensitive to inhibition of glycolytic and glutaminolytic enzymes. This was demonstrated by using lactate dehydrogenase A (LDHA) as an example. He further reported that survival in a transgenic model of MYC-dependent liver cancer could be prolonged by treatment with BPTES, an inhibitor of glutaminase.

Pharmaceutical efforts to target mutant IDH already provide clinical proof of concept that acute myelogenous leukaemia could be treated in humans in phase I studies. Katharine Yen (Agios) showed that targeting mutant IDH can provide clinical benefit. Mutant IDH1/2 drives 2HG accumulation, leading to histone and DNA hypermethylation, suppressing hematopoietic differentiation. Inhibitors of mutant IDH were able to reverse this hypermethylation and to induce differentiation in leukaemia models, resulting in significant survival benefit, in vivo.

Although cancer cells have been shown to use glucose and glutamine, alternative nutrient sources are less well understood. Eyal Gottlieb (Beatson Institute) discussed the role of acetate metabolism in hypoxic cancer cells. Hypoxia limits production of acetyl-CoA from glucose, which is largely converted to lactate through anaerobic glycolysis. By using siRNA screens, it was found that acetyl-CoA synthetase 2 (ACSS2), which catalyses the production of acetyl-CoA from acetate, was essential for cellular growth in hypoxic and nutrient-stressed conditions. ACSS2 is highly amplified in breast cancer, and further investigation confirmed the role of ACSS2 in driving acetate consumption for fatty acid synthesis. Silencing of ACSS2 suppresses cancer cell growth, both in vitro and in vivo. Thus, ACSS2 could be an attractive therapeutic target.

## Conclusion

The conference provided the audience a broad sampling of up-to-date knowledge and state-of-the-art technological developments. Next year’s Beatson Conference will be between the 5^th^ and 8^th^ of July, and its theme will be *Control of Cell Polarity and Movement in Cancer*.

