# Erratum: The 2014 Beatson international cancer conference: powering the cancer machine

**DOI:** 10.1186/s40170-014-0127-8

**Published:** 2014-12-23

**Authors:** Jurre J Kamphorst, Daniel J Murphy

**Affiliations:** Cancer Research UK Beatson Institute and Institute of Cancer Sciences, University of Glasgow, Garscube Estate, Switchback Road, Glasgow, G61 1BD UK

## Erratum

After publication of this Meeting Report [1], it emerged that sufficient permissions had not been granted to publish a particular section of the article. The relevant text has been replaced with:

Anne Brunet (Stanford University) discussed the metabolic regulation of aging using the C. elegans model. She focused on histone methylation as deficiencies in trimethylation of histone 3 at lysine 4 (H3K4me3) were found to increase lifespan. Regulation of H3K27me3 was also involved in lifespan determination. Brunet further discussed the importance of fat metabolism, in particular the different type of fatty acids, in H3K4me3-deficient worms and how this relates to longevity.

We apologise for any inconvenience.
